# Smoking and smoking cessation in relation to risk of diabetes in Chinese men and women: a 9-year prospective study of 0·5 million people

**DOI:** 10.1016/S2468-2667(18)30026-4

**Published:** 2018-03-13

**Authors:** Xin Liu, Fiona Bragg, Ling Yang, Christiana Kartsonaki, Yu Guo, Huaidong Du, Zheng Bian, Yiping Chen, Canqing Yu, Jun Lv, Kang Wang, Hua Zhang, Junshi Chen, Robert Clarke, Rory Collins, Richard Peto, Liming Li, Zhengming Chen, China Kadoorie Biobank Collaborative Group, Junshi Chen, Zhengming Chen, Robert Clarke, Rory Collins, Yu Guo, Liming Li, Jun Lv, Richard Peto, Robin Walters, Daniel Avery, Derrick Bennett, Ruth Boxall, Fiona Bragg, Yumei Chang, Yiping Chen, Huaidong Du, Simon Gilbert, Alex Hacker, Michael Holmes, Christiana Kartsonaki, Rene Kerosi, Garry Lancaster, Kuang Lin, John McDonnell, Iona Millwood, Qunhua Nie, Jayakrishnan Radhakrishnan, Paul Ryder, Sam Sansome, Dan Schmidt, Rajani Sohoni, Becky Stevens, Iain Turnbull, Robin Walters, Jenny Wang, Lin Wang, Neil Wright, Ling Yang, Xiaoming Yang, Zheng Bian, Ge Chen, Xiao Han, Can Hou, Pei Pei, Shuzhen Qu, Yunlong Tan, Canqing Yu, Zengchang Pang, Ruqin Gao, Shaojie Wang, Yongmei Liu, Ranran Du, Yajing Zang, Liang Cheng, Xiaocao Tian, Hua Zhang, Silu Lv, Junzheng Wang, Wei Hou, Jiyuan Yin, Ge Jiang, Xue Zhou, Liqiu Yang, Hui He, Bo Yu, Yanjie Li, Huaiyi Mu, Qinai Xu, Meiling Dou, Jiaojiao Ren, Shanqing Wang, Ximin Hu, Hongmei Wang, Jinyan Chen, Yan Fu, Zhenwang Fu, Xiaohuan Wang, Min Weng, Xiangyang Zheng, Yilei Li, Huimei Li, Yanjun Wang, Ming Wu, Jinyi Zhou, Ran Tao, Jie Yang, Chuanming Ni, Jun Zhang, Yihe Hu, Yan Lu, Liangcai Ma, Aiyu Tang, Shuo Zhang, Jianrong Jin, Jingchao Liu, Zhenzhu Tang, Naying Chen, Ying Huang, Mingqiang Li, Jinhuai Meng, Rong Pan, Qilian Jiang, Weiyuan Zhang, Yun Liu, Liuping Wei, Liyuan Zhou, Ningyu Chen, Hairong Guan, Xianping Wu, Ningmei Zhang, Xiaofang Chen, Xuefeng Tang, Guojin Luo, Jianguo Li, Xunfu Zhong, Jiaqiu Liu, Qiang Sun, Pengfei Ge, Xiaolan Ren, Caixia Dong, Hui Zhang, Enke Mao, Xiaoping Wang, Tao Wang, Xi Zhang, Ding Zhang, Gang Zhou, Shixian Feng, Liang Chang, Lei Fan, Yulian Gao, Tianyou He, Huarong Sun, Pan He, Chen Hu, Qiannan Lv, Xukui Zhang, Min Yu, Ruying Hu, Hao Wang, Yijian Qian, Chunmei Wang, Kaixue Xie, Lingli Chen, Yidan Zhang, Dongxia Pan, Yuelong Huang, Biyun Chen, Li Yin, Donghui Jin, Huilin Liu, Zhongxi Fu, Qiaohua Xu, Xin Xu, Hao Zhang, Youping Xiong, Huajun Long, Xianzhi Li, Libo Zhang, Zhe Qiu

**Affiliations:** a Department of Epidemiology and Health Statistics, School of Public Health, Xi'an Jiaotong University Health Science Center, Xi'an, China; b Clinical Trial Service Unit and Epidemiological Studies Unit, Nuffield Department of Population Health, University of Oxford, Oxford, UK; c Medical Research Council Population Health Research Unit, Nuffield Department of Population Health, University of Oxford, Oxford, UK; d Chinese Academy of Medical Sciences, Beijing, China; e Department of Epidemiology and Biostatistics, School of Public Health, Peking University Health Science Center, Beijing, China; f Shibei Centre for Disease Control and Prevention, Qingdao, China; g Qingdao Centre for Disease Control and Prevention, Qingdao, China; h National Center for Food Safety Risk Assessment, Beijing, China

## Abstract

**Background:**

In developed countries, smoking is associated with increased risk of diabetes. Little is known about the association in China, where cigarette consumption has increased (first in urban, then in rural areas) relatively recently. Moreover, uncertainty remains about the effect of smoking cessation on diabetes in China and elsewhere. We aimed to assess the associations of smoking and smoking cessation with risk of incident diabetes among Chinese adults.

**Methods:**

The prospective China Kadoorie Biobank enrolled 512 891 adults (59% women) aged 30–79 years during 2004–08 from ten diverse areas (five urban and five rural) across China. Participants were interviewed at study assessment clinics, underwent physical measurements, and had a non-fasting blood sample taken. Participants were separated into four categories according to smoking history: never-smokers, ever-regular smokers, ex-smokers, and occasional smokers. Incident diabetes cases were identified through linkage with diabetes surveillance systems, the national health insurance system, and death registries. All analyses were done separately in men and women and Cox regression was used to yield adjusted hazards ratios (HRs) for diabetes associated with smoking.

**Findings:**

68% (n=134 975) of men ever smoked regularly compared with 3% (n=7811) of women. During 9 years' follow-up, 13 652 new-onset diabetes cases were recorded among 482 589 participants without previous diabetes. Among urban men, smokers had an adjusted HR of 1·18 (95% CI 1·12–1·25) for diabetes. HRs increased with younger age at first smoking regularly (1·12, 1·20, and 1·27 at ≥25 years, 20–24 years, and <20 years, respectively; p for trend=0·00073) and with greater amount smoked (1·11, 1·15, 1·42, and 1·63 for <20, 20–29, 30–39 and ≥40 cigarettes per day; p for trend<0·0001). Among rural men, similar, albeit more modest, associations were seen. Overall, HRs were more extreme at higher levels of adiposity. Among men who stopped by choice, there was no excess risk within 5 years of cessation, contrasting with those who stopped because of illness (0·92 [0·75–1·12] *vs* 1·42 [1·23–1·63]). Among the few women who ever smoked regularly, the excess risk of diabetes was significant (1·33 [1·20–1·47]).

**Interpretation:**

Among Chinese adults, smoking was associated with increased risk of diabetes, with no significant excess risk following voluntary smoking cessation.

**Funding:**

Wellcome Trust, Medical Research Council, British Heart Foundation, Cancer Research UK, Kadoorie Charitable Foundation, Ministry of Science and Technology, National Natural Science Foundation of China, and China Scholarship Council.

## Introduction

Globally, an estimated 425 million people have diabetes, causing a substantial burden of premature death and disability.^1^ In China, the prevalence of diabetes has increased dramatically over recent decades, from approximately 1% in 1980,^2^ to around 11% in 2013.^3^ However, the factors underlying this escalating prevalence are incompletely understood. Apart from increasing adiposity,^4^ other lifestyle factors, including smoking, might play a part.^5^ In China, cigarette consumption has increased markedly over a similar time period, first in urban areas, then in rural areas, although this increase has occurred almost exclusively in men.^6^ About two-thirds of Chinese men now smoke,^6^ consuming roughly 40% of the world's cigarettes.^7^ A few prospective studies^8^, ^9^ have examined the association between smoking and diabetes in China, but these were constrained by small sample size, short follow-up (usually <5 years), insufficiently detailed information on smoking patterns, and restriction to particular urban populations. As such, substantial uncertainty remains about the associations of smoking and smoking cessation with diabetes in China.

Accumulating evidence, mainly from developed countries, suggests a positive dose-response association of smoking with risk of type 2 diabetes.^10^, ^11^ Questions persist, however, about the causal nature of the observed associations, about the effects of smoking cessation on diabetes risk, and about the potential role of adiposity—a major cause of diabetes^4^—in affecting the association. While smokers tend to have lower adiposity than non-smokers, heavy smokers tend to have higher abdominal fat accumulation than light or non-smokers.^12^ Moreover, smoking cessation can lead to weight gain, with previous studies^10^ reporting elevated risk of diabetes within a few years of stopping. While weight gain could possibly account for this association,^12^ the excess risk might also be due to reverse causality, since a proportion of ex-smokers might have stopped because of poor health. However, previous studies have lacked the detailed information (eg, specific reasons for stopping) to clarify this association. Reliable elucidation of the associations of smoking and smoking cessation with diabetes will inform tobacco control strategies.

**Research in context**

**Evidence before this study**

We searched PubMed for articles published in English up to Aug 31, 2017, using the search terms “smoking”, “diabetes”, “prospective”, and “cohort”. Four relevant meta-analyses were identified, which all found positive associations of current regular smoking with risk of incident diabetes (pooled hazard ratios ranging from 1·37 to 1·44), and higher risk with greater number of cigarettes smoked. One meta-analysis, including ten prospective studies, also examined the association with smoking cessation, showing that individuals who had stopped for less than 5 years had significantly elevated risk, which decreased as time since cessation increased. These meta-analyses included populations from predominantly developed countries, where widespread cigarette smoking among young adults has persisted for several decades. In 2014, about 40% of the world's cigarettes were consumed in China, almost exclusively by men, but the increase in cigarette usage, first urban then rural, has been relatively recent. Two prospective studies in China, which recruited participants between 2000 and 2006, have also examined the association between smoking and diabetes. However, these studies included relatively small sample sizes (3598 and 51 464) and few diabetes cases (160 and 1304), with inconsistent findings.

**Added value of this study**

Our aim was to assess the associations of smoking and smoking cessation with risk of incident diabetes among urban and rural Chinese adults. We showed that smoking is associated with a higher risk of diabetes among men and women. Dose–response effects were clear with age at first starting to smoke regularly and with amount smoked. Among men, the relative risks were more extreme in urban areas than in rural areas. Given the amount smoked, the relative risks appeared somewhat greater among women than men. We also found strong evidence that high body fat content might amplify the excess risk associated with smoking among men. Furthermore, stopping smoking by choice (ie, before development of serious illness) was not associated with excess risk of developing diabetes in the first 5 years of cessation, in contrast with the higher risk among those who stopped because of illness.

**Implications of all the available evidence**

Smoking is an important modifiable risk factor for diabetes in diverse populations. Further studies, including Mendelian randomisation analyses, might enable investigation of the causality of the association. Irrespective of causality, smoking should be targeted as an important lifestyle factor in disease prevention strategies, including for diabetes, in China and elsewhere.

Using data from the large nationwide China Kadoorie Biobank (CKB) prospective study, we evaluate prospective associations of smoking and smoking cessation with risk of incident diabetes, and explore potential effect modification by adiposity.

## Methods

### Study population

Details of the CKB study design and methods have been described previously.^13^, ^14^ The CKB is a prospective cohort study of 512 891 (210 259 men and 302 632 women) adults recruited from ten areas (five urban and five rural) of China. These areas were chosen from China's nationally representative Disease Surveillance Points to ensure coverage of a range of socioeconomic levels, disease patterns, and risk factors. Between June 25, 2004, and July 15, 2008, all registered residents aged 35–74 years were invited to participate in the baseline survey; approximately 30% responded, including about 10 000 participants slightly outside the target age range (making the actual baseline age range from 30 years to 79 years).

At study assessment clinics, participants were interviewed by trained health workers, using laptop-based questionnaires, on sociodemographic status, lifestyle (eg, smoking, alcohol consumption, diet, and physical activity), and medical history. Anthropometric measurements (eg, height, weight, and waist and hip circumferences) were done using calibrated instruments with standard protocols, with participants wearing light clothing and no shoes. Weight was measured using a body composition analyser (TANITA-TBF-300GS; Tanita Corporation, Tokyo, Japan), which also measured body fat percentage with its inbuilt proprietary algorithm based on foot-to-foot bioelectrical impedance. Standing height was obtained to the nearest 0·1 cm with a stadiometer. Body-mass index (BMI) was calculated as weight in kilograms divided by the square of standing height in metres (kg/m^2^). Waist and hip circumferences were measured using a non-stretchable tape to the nearest 0·1 cm. Systolic blood pressure and diastolic blood pressure were measured in a seated position after at least 5 min rest using a digital sphygmomanometer (Omron UA-779; A&D Instruments, Abingdon, UK). A 10 mL non-fasting blood sample (with time since last meal recorded) was collected into an EDTA vacutainer for storage and onsite random plasma glucose testing using the SureStep Plus system (LifeScan, Milpitas, CA, USA). Two resurveys (from May 26 to Oct 10, 2008, and from Aug 4, 2013, to Sept 18, 2014) including a randomly selected sample of approximately 5% of participants were undertaken, collecting the same information as at baseline. Ethics approval was obtained from the Oxford University Tropical Research Ethics Committee and the Chinese Center for Disease Control and Prevention Ethical Review Committee.

### Assessment of smoking

Smoking information collected included frequency, amount, and type of tobacco smoked, currently and in the past, and the ages at which participants started smoking regularly and stopped smoking. Among current and former (or ex) regular smokers, additional information was collected on the types and amount of tobacco smoked when last smoking, with amount calculated in g/day, assuming 1 g of tobacco per factory cigarette, 2 g per cigar, and actual amount in pipes and hand-rolled cigarettes as reported. Smoking duration was derived from the age at starting smoking regularly to age at baseline (current smokers) or duration since quitting at baseline (ex-smokers). Among individuals who reported having stopped smoking, the main reason for smoking cessation (physical illness or by choice) was collected. Four categories of smoking status were defined: never-smokers (participants who reported not smoking at recruitment and who had smoked <100 cigarettes during their lifetime); ever-regular smokers (participants who reported ever smoking at least one cigarette or 1 g tobacco per day for ≥6 months, or who had stopped smoking ≥6 months before recruitment because of ill health [included in this group to avoid bias]); ex-smokers by choice (participants who stopped smoking ≥6 months before recruitment by choice [providing information on the effects of smoking cessation]); and occasional smokers (participants who did not meet the criteria for never-smoker or ever-regular smoker, and who had not stopped smoking completely for at least the 6 months before recruitment). Pack-years were not examined since smoking ten cigarettes per day for 30 years might have different effects than smoking 20 cigarettes per day for 15 years.^15^ Exhaled carbon monoxide was measured to validate smoking exposure (CareFusion MicroCO meter; CareFusion, Chatham, UK).

### Follow-up for incident diabetes

Cause-specific morbidity and mortality were monitored through linkage, via unique national identification number, with disease (including diabetes) and death registries, and with the national health insurance system, which has almost universal coverage (>98%) across the ten study areas. Annual active follow-up was performed by checking against local residential records to confirm survival status and to minimise losses to follow-up. Causes of death were classified on the basis of official death certificates and were checked against available medical records when necessary. Linkage to the health insurance system enabled identification of diagnoses resulting in, or during, hospital admissions. Incident diabetes cases were identified through the diabetes disease surveillance system and through diabetes diagnoses (ICD-10 E10-E14 codes) recorded in health insurance databases or underlying or contributing to death on death certificates.

### Statistical analysis

In this study, participants with self-reported diabetes that was clinically diagnosed, screen-detected diabetes (no self-reported diabetes and a plasma glucose concentration ≥7·0 mmol/L and a fasting time ≥8 h, a plasma glucose concentration ≥11·1 mmol/L and a fasting time <8 h, or a fasting plasma glucose concentration ≥7·0 mmol/L),^16^ or with missing BMI data at baseline were excluded. Sensitivity analyses further excluded participants with a history of cancer or cardiovascular disease at baseline, and, separately, participants who developed diabetes, died, or were lost to follow-up during the first 3 years of follow-up.

All analyses were done separately in men and women. Prevalence and mean values of characteristics at baseline were calculated across smoking categories standardised by age (5-year age groups) and study area, when appropriate. Cox regression was used to estimate adjusted hazard ratios (HRs) of diabetes, with time since entry into the study as the underlying timescale. Participants contributed person-years until diagnosis of diabetes, death, loss to follow-up, or the censoring date (Dec 31, 2015). Models were stratified by age at risk (5-year groups) and study area, and adjusted for educational attainment (no formal education, primary school, middle or high school, or college or university and above), alcohol consumption (never, occasional, or ever-regular), and physical activity as metabolic equivalent of task hours per day (model 1), and further adjusted for BMI and waist circumference as continuous variables (model 2).

The main analyses were done separately in urban areas and rural areas because of well documented differences in their past smoking patterns.^6^ χ^2^ tests for trend and heterogeneity were applied to the log HRs and their SEs.^17^ The floating absolute risk method was used; this method does not alter the value of the HRs but provides a 95% CI for each HR, derived from the variance of the log risk in that category, enabling comparisons between any two categories and not only with the reference group.^18^ Incidence rates per 1000 person-years (R_i_) were calculated from the adjusted HRs using a weighted method with the number of events in each group as the weighting variable, W_i_, using the formula:

$${\text{R}}_{i}={\text{HR}}_{i}\times \frac{\text{overall}\;\text{rate}}{\left(\frac{\left[\sum {\text{W}}_{i}\times {\text{HR}}_{i}\right]}{\text{total}\;\text{number}\;\text{of}\;\text{events}}\right)}$$

Analyses were done using SAS version 9.3 and R version 3.3.2.

### Role of the funding source

The funders of the study had no role in the design of the study, collection, analysis, or interpretation of data, or in the writing of the report. FB and ZC had full access to the data and responsibility for the final decision to submit for publication.

## Results

16 162 participants with self-reported diabetes that was clinically diagnosed, 14 138 participants with screen-detected diabetes, and two with missing BMI data at baseline were excluded, leaving 482 589 participants (198 574 men and 284 015 women) for the main analyses. Sensitivity analyses further excluded 21 376 participants with a history of cancer or cardiovascular disease at baseline, and, separately, 2760 participants who developed diabetes, died, or were lost to follow-up during the first 3 years of follow-up were excluded. By Jan 1, 2016, 37 289 (7·3%) participants had died, with only 4875 (<1%) lost to follow-up.

Overall 68% (n=134 975) of men (62% [n=51 990] urban and 72% [n=82 985] rural) and 3% (n=7811) of women (2% [n=2772] urban and 3% [n=5039] rural) were ever-regular smokers. Among men, ever-regular smokers were younger (p<0·0001), and ex-smokers older (p<0·0001), than never-smokers (table 1). Ever-regular smokers seemed more likely than never-smokers to have lower educational attainment, be regular alcohol or tea drinkers, and consume more meat, but less fresh fruit (table 1). Among ever-regular smokers, there was a positive dose-dependent association of amount smoked with waist circumference—a marker of central adiposity—independent of general adiposity, but a less clear association with general adiposity (BMI or body fat percentage; appendix p 7). Blood pressure was lower among ever-regular smokers and higher among ex-smokers, compared with never-smokers (table 1). Mean exhaled carbon monoxide was much higher among current regular smokers (15·4 ppm [SD 10·6]; ie, ever-regular smokers excluding ex-smokers who stopped because of illness) than among participants in other smoking categories, while random plasma glucose concentrations did not differ across smoking categories (table 1).

**Table 1:** Baseline characteristics for men by smoking status

	**Never-smokers (n=28 214)**	**Ex-smokers (n=12 950)**	**Occasional smokers (n=22 435)**	**Ever-regular smokers (n=134 975)**
Age (years)	53·4 (11·9)	55·3 (10·9)	50·0 (11·1)	51·7 (10·5)
Urban residence	15 278 (53·7%)	6715 (49·9%)	9735 (43·4%)	51 990 (38·5%)
Education >6 years	18 622 (60·5%)	7629 (59·3%)	14 834 (60·6%)	73 035 (55·6%)
Exhaled carbon monoxide (ppm)	5·0 (5·5)	5·3 (5·5)	5·6 (5·0)	14·1 (9·5)
Random plasma glucose (mmol/L)*	5·6 (1·3)	5·6 (1·3)	5·6 (1·2)	5·6 (1·2)
Systolic blood pressure (mm Hg)	133·1 (20·3)	134·2 (20·0)	132·5 (20·5)	132·0 (19·5)
Diastolic blood pressure (mm Hg)	79·6 (12·1)	80·4 (12·2)	79·4 (11·9)	78·8 (11·3)
Weight (kg)	64·6 (10·7)	66·4 (10·8)	64·8 (10·2)	63·4 (9·8)
Height (cm)	164·8 (6·6)	165·5 (6·5)	164·9 (6·1)	165·3 (5·8)
BMI (kg/m^2^)	23·7 (3·4)	24·2 (3·3)	23·7 (3·2)	23·1 (3·1)
Waist circumference (cm)	82·1 (10·1)	84·0 (10·1)	82·4 (9·5)	81·3 (9·3)
Waist-hip ratio	0·9 (0·1)	0·9 (0·1)	0·9 (0·1)	0·9 (0·1)
Body fat percentage (%)†	22·3 (6·5)	22·3 (6·2)	23·3 (6·5)	21·6 (5·9)
Physical activity (MET h/day)	22·1 (15·5)	22·2 (14·9)	22·9 (13·8)	22·3 (13·2)
Regular alcohol drinker	6627 (24·3%)	6213 (45·8%)	6719 (31·9%)	63 371 (46·8%)
Regular tea drinker	10 166 (35·3%)	6184 (50·4%)	8170 (40·3%)	76 319 (55·3%)
Regular meat consumption	15 356 (48·5%)	7013 (52·3%)	11 022 (49·2%)	68 106 (51·9%)
Regular fresh fruit consumption	9310 (28·3%)	3944 (26·9%)	5609 (26·1%)	26 368 (20·5%)
Family history of diabetes	1826 (6·2%)	789 (5·7%)	1472 (6·2%)	7898 (5·9%)

Values are mean (SD) or number of participants (%), standardised to age and study area structure of study population. Ex-smokers=ex-smokers who stopped by choice. Ever-regular smokers=current smokers and ex-smokers who stopped because of illness. BMI=body-mass index. MET h=metabolic equivalent of task hours. Regular alcohol drinker=current or previous consumption at least once weekly. Regular tea drinker=consumption at least once weekly. Regular meat consumption=consumption on at least 4–6 days per week. Regular fruit consumption=consumption on at least 4–6 days per week.

* Data missing for 301 never-smokers, 229 ex-smokers, 338 occasional smokers, and 2426 ever-regular smokers.

† Data missing for 15 never-smokers, six ex-smokers, ten occasional smokers, and 85 ever-regular smokers.

During roughly 9 years' follow-up (4·3 million person-years), 5194 men and 8458 women developed new-onset diabetes among 482 589 participants without previous diabetes, with incidence rates of 3·0 per 1000 person-years for men and 3·3 per 1000 person-years for women. Smoking was associated with higher risk of diabetes, with adjusted HRs of 1·15 (95% CI 1·05–1·27) for ex-smokers (stopped by choice), 1·11 (1·02–1·21) for occasional smokers, and 1·04 (1·00–1·08) for ever-regular smokers, among men (table 2). The HRs were greater in urban areas than in rural areas (table 2). After further adjustment for BMI and waist circumference, the risks were substantially attenuated in ex-smokers (1·05, 0·96–1·16), but increased in ever-regular smokers (1·14, 1·10–1·18; table 2). The associations were not materially changed by further excluding individuals with a history of cancer or cardiovascular disease at baseline, or individuals who developed incident diabetes during the first 3 years of follow-up, or by further adjusting for dietary factors (appendix p 2) or age at baseline as a continuous variable.

**Table 2:** Adjusted HR of incident diabetes in men according to smoking status

	**Urban**				**Rural**				**Overall**			
	Cases (n)	Rate*	HR (95% CI)		Cases (n)	Rate*	HR (95% CI)		Cases (n)	Rate*	HR (95% CI)	
			Model 1	Model 2			Model 1	Model 2			Model 1	Model 2
Never-smokers	400	2·80	1·00 (0·90–1·11)	1·00 (0·90–1·11)	319	2·60	1·00 (0·89–1·12)	1·00 (0·89–1·12)	719	2·68	1·00 (0·93–1·08)	1·00 (0·93–1·08)
Ex-smokers	229	3·11	1·21 (1·06–1·38)	1·11 (0·98–1·27)	201	2·59	1·09 (0·95–1·25)	1·00 (0·87–1·14)	430	2·82	1·15 (1·05–1·27)	1·05 (0·96–1·16)
Occasional smokers	268	3·07	1·10 (0·98–1·25)	1·09 (0·97–1·24)	291	2·96	1·11 (0·99–1·24)	1·14 (1·01–1·28)	559	3·00	1·11 (1·02–1·21)	1·12 (1·03–1·21)
Ever-regular smokers	1426	3·31	1·11 (1·05–1·18)	1·18 (1·12–1·25)	2060	2·87	0·97 (0·93–1·02)	1·10 (1·05–1·15)	3486	3·05	1·04 (1·00–1·08)	1·14 (1·10–1·18)
p for heterogeneity	..	..	0·14	0·045	..	..	0·12	0·20	..	..	0·061	0·019

Model 1 was stratified by age at risk and study area and adjusted for education, alcohol consumption, and physical activity. Model 2 was additionally adjusted for body-mass index and waist circumference. HR=hazard ratio. Ex-smokers=ex-smokers who stopped by choice. Ever-regular smokers=current smokers and ex-smokers who stopped because of illness.

* Incidence rate per 1000 person-years was calculated from the HRs (model 2) using a weighted method with the number of events in each group as the weighting variable.

**Figure 1:**
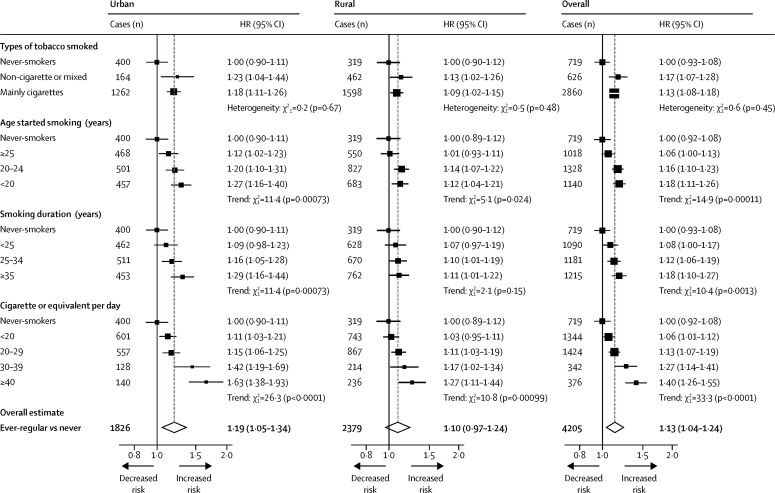
Adjusted HRs of incident diabetes by smoking in men Stratified by age at risk and study area and adjusted for education, alcohol consumption, physical activity, body-mass index, and waist circumference. Analyses examining smoking duration were additionally adjusted for age at baseline. Ever-regular smokers excludes occasional smokers (n=22 435) and ex-smokers who stopped by choice (n=12 950). Tests for trend include all smoking categories. Tests for heterogeneity include only smokers. HR=hazard ratio.

Among urban men, the HRs differed little by tobacco type, but were higher among those who started smoking at an earlier age (1·12, 1·20, and 1·27 at ≥25 years, 20–24 years, and <20 years, respectively; p for trend=0·00073; figure 1). Similarly, the HRs increased with increasing duration of smoking (p for trend=0·00073; figure 1), and amount smoked (p for trend <0·0001; figure 1), with a HR of 1·52 (95% CI 1·35–1·72) among those who smoked 30 cigarettes or more per day. The patterns of association were similar among current regular smokers (appendix p 8). Similar associations, albeit more modest, were observed among rural men (figure 1), with no significant heterogeneity across individual rural (p for heterogeneity=0·28) or urban (p for heterogeneity=0·57) study areas (appendix p 9). Similarly, the HRs for ever-regular smoking did not vary markedly across different population subgroups (eg, by age, education, physical activity, alcohol intake, and systolic blood pressure; appendix p 10).

**Figure 2:**
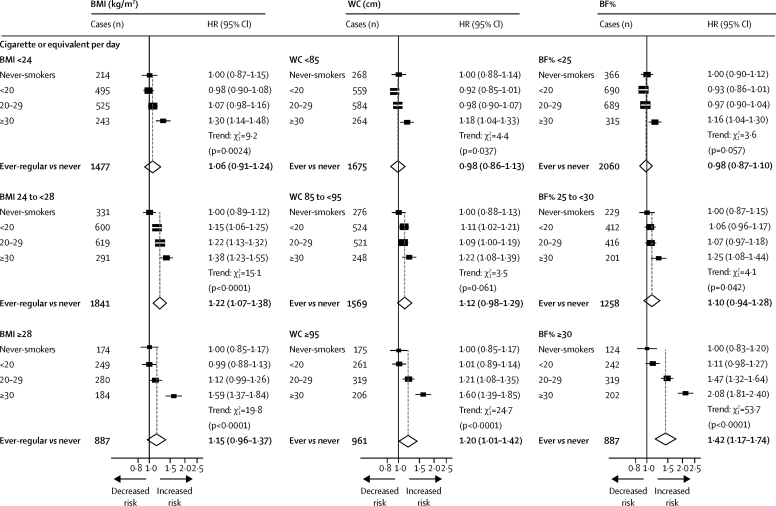
Adjusted HRs of incident diabetes by amount smoked, stratified by levels of adiposity, in men Stratified by age at risk and study area and adjusted for education, alcohol consumption, and physical activity. Ever-regular smokers excludes occasional smokers and ex-smokers who stopped by choice. HR=hazard ratio. BMI=body-mass index. WC=waist circumference. BF%=body fat percentage.

When stratified by levels of adiposity, the HRs associated with smoking amount appeared stronger among men with higher levels of adiposity (figure 2, appendix p 3). Compared with never-smokers, the HRs for those who smoked 30 cigarettes or more per day were 1·18 (95% CI 1·04–1·33), 1·22 (1·08–1·39), and 1·60 (1·39–1·85) among those with waist circumferences of less than 85 cm, 85 cm to less than 95 cm, and 95 cm or more, respectively (figure 2; p for trend=0·0016). Similar patterns were observed across strata of body fat percentage (p for trend <0·0001) and BMI (figure 2), although with a less extreme trend (p for trend=0·048).

**Figure 3:**
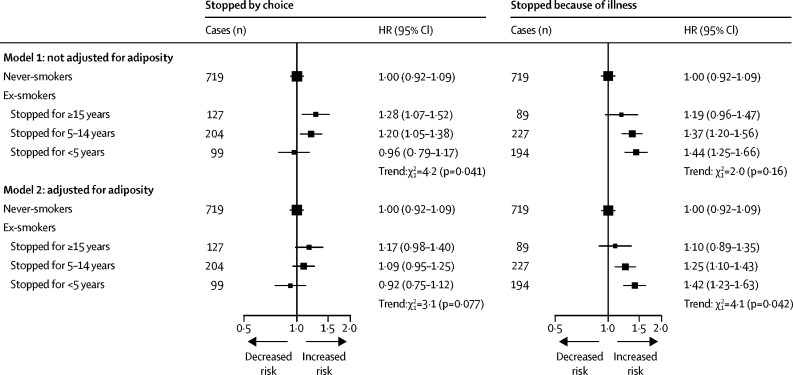
Adjusted HRs of incident diabetes in male ex-smokers by reasons for, and years after, smoking cessation Model 1 was stratified by age at risk and study area and adjusted for education, alcohol consumption, and physical activity. Model 2 was additionally adjusted for body-mass index and waist circumference. Tests for trend include only ex-smokers. HR=hazard ratio.

Among men who stopped smoking because of illness, the excess risk of diabetes was significant, which attenuated with increasing time since quitting: HRs were 1·44 (95% CI 1·25–1·66), 1·37 (1·20–1·56), and 1·19 (0·96–1·47) for those who had stopped for less than 5 years, 5–14 years, and 15 years or more before baseline, respectively (p for trend=0·16; figure 3). Consideration of time since smoking cessation as a time-updating variable showed similar results (appendix p 11). Additional adjustment for BMI and waist circumference slightly attenuated these associations (figure 3). By contrast, among ex-smokers who stopped by choice, the HRs tended to increase with longer time since quitting, with HRs of 0·96 (0·79–1·17), 1·20 (1·05–1·38) and 1·28 (1·07–1·52) among those who had stopped for less than 5 years, 5–14 years, and 15 years or more before baseline (p for trend=0·041; figure 3). However, these associations were attenuated after further adjustment for BMI and waist circumference (p for trend=0·077).

**Figure 4:**
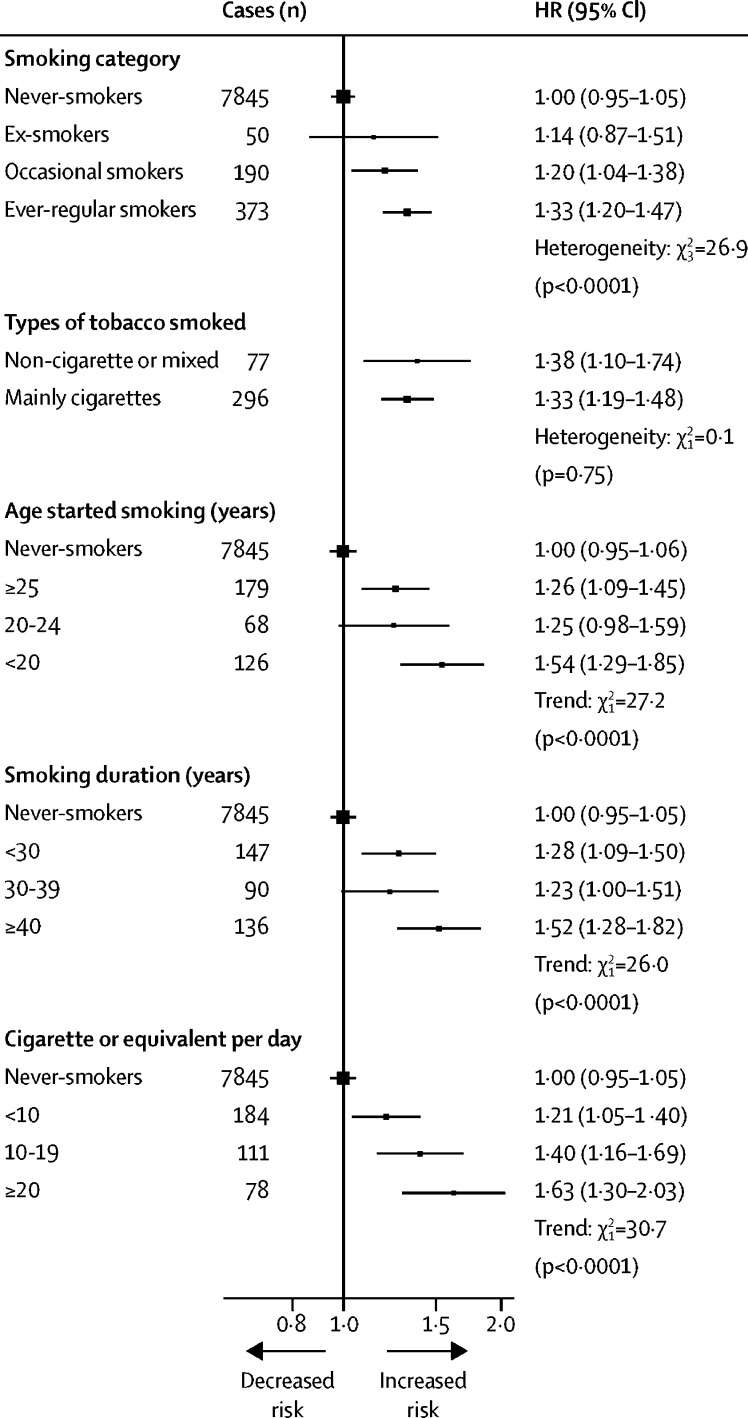
Adjusted HRs of incident diabetes by smoking in women Stratified by age at risk and study area and adjusted for education, alcohol consumption, physical activity, body-mass index, and waist circumference. Analyses examining smoking duration were additionally adjusted for age at baseline. Tests for trend include all smoking categories. Test for heterogeneity across smoking categories includes all smoking categories. Other tests for heterogeneity include only smokers. HR=hazard ratio.

Among women, ever-regular smokers were, on average, older, and had higher adiposity, than never-smokers (appendix p 4). Compared with never-smokers, the HR of diabetes was 1·33 (95% CI 1·20–1·47; figure 4), which was somewhat more extreme than that among men (p for heterogeneity=0·0049). The adjusted HRs by tobacco category, age of starting smoking, duration of smoking, and daily amount smoked were broadly similar to those in men, with women who smoked more than 20 cigarettes per day having a HR of 1·63 (1·30–2·03; figure 4). By contrast with men, adiposity did not appear to modify the risk associated with smoking among women, but this might reflect inadequate power to examine such associations (appendix p 12).

## Discussion

In this large, nationwide, prospective cohort study, smoking was associated in a dose-dependent manner with increased risk of incident diabetes among men and women. Among men, the relative risk appeared stronger among individuals with higher adiposity. Given the amount smoked, women tended to have somewhat greater relative risk than men. Furthermore, after allowing for adiposity, smoking cessation was associated with an elevated risk of diabetes only among men who stopped smoking because of illness, and not those who stopped by choice.

A 2015 meta-analysis^10^ of published data from 84 prospective studies, including almost 300 000 diabetes cases, reported an overall HR of diabetes of 1·37 (95% prediction interval 1·11–1·70), associated with active smoking, with a non-significantly stronger association among men (1·42) than women (1·33). Most previous studies^10^, ^11^, ^19^, ^20^, ^21^, ^22^ have been in high-income countries, where, by contrast with China, persistent use of cigarettes among young adults has lasted for a longer period of time. Before the 1970s, total cigarette consumption in China was low and stable. Since the early 1980s, however, cigarette consumption has increased five-fold, almost exclusively among men (as shown by the National Bureau of Statistics of China). In China, although more than two-thirds of men smoke^6^ and more than 10% of adults are affected by diabetes,^3^ evidence on the association of smoking with diabetes is sparse and inconsistent. In the Shanghai Men's Health study,^9^ with 1304 incident diabetes cases (identified through self-report) after 5 years of follow-up, the increased risk of diabetes was evident among men who smoked 20 cigarettes or more per day (HR 1·25, 95% CI 1·00–1·56), but not among men who smoked less. By contrast, a small prospective study^8^ in China, including only 160 diabetes cases reported an overall HR of 4·16 (2·77–6·24) among current smokers. Our study included almost ten times more diabetes cases than previous studies in China combined, and demonstrated a modest, but clear, positive dose-dependent association of smoking with risk of diabetes among men and women.

Unlike in developed populations, where tobacco use is almost exclusively in the form of cigarettes, a high proportion of smokers in China (especially those born before the 1970s in rural areas) smoke non-cigarette tobacco (eg, pipes and water pipes).^6^ However, the present study showed that the risk of diabetes did not differ significantly by tobacco type. Total amount of tobacco smoked did not differ between cigarette (18·3 cigarettes or equivalents per day) and non-cigarette or mixed smokers (18·1 cigarettes or equivalents per day), nor in the proportions reporting inhaling deeply into the lungs (76% *vs* 73%), suggesting that tobacco dose, rather than type, might be more relevant for risk of diabetes. Overall, our relative risk estimates were more modest than those reported previously in developed populations and were less extreme among men from rural areas than from urban areas, consistent with previous findings showing that the tobacco epidemic is still at an early stage in China, particularly in rural areas.^6^ Cigarette consumption became widespread earlier in urban, than in rural, areas in China,^6^ but with rapid economic development, cigarettes have become more readily available and affordable in rural areas.^23^, ^24^ An upward trend in diabetes risk attributable to smoking would, therefore, be expected in rural areas, unless there is widespread smoking cessation.

Obesity is a well recognised modifiable risk factor for diabetes.^4^ Smokers, on average, tend to be leaner than non-smokers, possibly reflecting appetite suppression and elevated resting metabolic rate associated with smoking.^12^ However, heavy smokers are more likely to have higher abdominal adiposity than light or non-smokers, as shown in previous studies^12^ and in the CKB. These adiposity associations could help to explain the excess diabetes risk among smokers, despite their relatively low average BMI. Previous evidence in developed populations has been conflicting, however, with regards to the interaction of adiposity and smoking on diabetes risk.^8^, ^19^, ^20^, ^21^, ^22^ Although two studies,^19^, ^20^ each with approximately 3000 diabetes cases, showed no such effect modification by BMI, a larger study,^22^ with approximately 12 000 incident cases, reported a stronger association among individuals with lower BMI, contrary to the present and another Chinese study's findings.^8^ Only two small studies^20^, ^21^ have examined the interaction of smoking amount with adiposity, showing inconsistent findings. In the present study, we observed a stronger association of amount smoked with risk of diabetes among individuals with higher adiposity, especially as assessed by body fat percentage. This observation might help to explain the greater HRs among women than men for a given amount smoked, because women have a greater proportion of body fat compared with men. The precise mechanism underlying this interaction is not clear, but animal studies have shown release of fatty acids from adipose tissue in response to nicotine administration, which might, in turn, promote insulin resistance^25^ and development of diabetes.

A meta-analysis^10^ pooling published data from ten prospective studies reported that individuals who stopped smoking more recently (<5 years) had a higher risk of diabetes than those who had stopped for a longer duration. Some smokers are likely to have quit because of poor health (including development of diabetes), resulting in a spurious association, but information about reasons for quitting smoking was not available in these previous studies. This assertion is supported by data in the CKB, in which individuals who stopped smoking because of illness tended to have higher baseline prevalence of cardiovascular diseases, hypertension, and diabetes than those who stopped by choice and than never-smokers (appendix p 5). Moreover, the present study showed that the excess risk among recent quitters was evident among individuals who had stopped smoking because of illness but not among those who stopped by choice, suggesting that the previously reported findings were due chiefly to reverse causality. Among individuals who stopped smoking by choice, excess risk was small among those who had stopped for longer periods of time (≥5 years), which could be attributable (at least partly) to weight gain following smoking cessation (appendix p 13), in keeping with attenuation of this excess risk after adjustment for adiposity. The interaction between adiposity and smoking is also likely to explain the contrasting effects of additional adjustment for adiposity on the risks of diabetes among ex-smokers (more adipose) and ever-regular smokers (less adipose).

In addition to the large study population, the availability of uniquely detailed information on smoking and smoking cessation enabled more comprehensive assessment of the associations of smoking with diabetes in the current study than has previously been possible. Detailed review of medical records of about 1000 randomly selected diabetes cases in CKB showed a high positive predictive value of diabetes diagnosis (97% based on American Diabetes Association diagnostic criteria^26^ and medication use). Furthermore, the diverse study population and extremely low loss to follow-up ensure generalisability of the findings and limit potential for biased risk estimates. There are, however, limitations of our study. First, without data on weight change after smoking cessation, it was not possible to assess the direct effect of this factor on the association of smoking cessation with diabetes. Second, potential confounding by total energy or specific nutrient consumption could not be assessed adequately. Third, a proportion of new-onset diabetes cases might not have been captured during follow-up. However, the two resurveys of a random subset of participants enabled estimation of the extent of undiagnosed diabetes, demonstrating that diabetes prevalence in the CKB was moderately lower than in contemporaneous nationally representative surveys, which used a combination of self-report, fasting blood glucose and glycated haemoglobin measurement, and oral glucose tolerance testing to detect diabetes (appendix p 6).^3^, ^27^ However, sensitivity analyses comparing the association of regular smoking with diagnosed and undiagnosed diabetes among men included in both resurveys showed no significant difference in the magnitude of association (p for heterogeneity 0·86). Fourth, type 1 diabetes cases might have been included in the analyses. However, given the age of the study population, the number would be expected to be extremely small and unlikely to affect the risk estimates. Finally, reliable investigation of the effects of smoking cessation on risk of diabetes and of differential association by adiposity among women was limited by the low prevalence of smoking among women in China.

Among men in China, the excess diabetes risk associated with smoking is likely to increase substantially in future generations because the tobacco epidemic is maturing and population mean adiposity is increasing.^28^ Encouragingly, despite increased risk of diabetes among female smokers, very few Chinese women now smoke, and stopping smoking before the onset of major illness could prevent the elevated risk of diabetes (and other diseases) associated with smoking. Irrespective of whether these associations are causal or not, smoking should be targeted as an important lifestyle factor in future disease prevention strategies, including for diabetes, in China and elsewhere.

For more on the **CKB** see http://www.ckbiobank.org/

For more on the **National Bureau of Statistics of China** see http://data.stats.gov.cn/
