# Supplementary material for: Smoking and smoking cessation in relation to risk of diabetes in Chinese men and women: a 9-year prospective study of 0·5 million people
Source: Lancet Public Health. 2018 Mar 13;3(4):e167–76. doi: 10.1016/S2468-2667(18)30026-4 (PMC5887081; doi:10.1016/S2468-2667(18)30026-4)

THE LANCET
Child & Adolescent Health

Supplementary appendix

This appendix formed part of the original submission and has been peer reviewed. We post it as supplied by the authors.

Supplement to: Liu X, Bragg F, Yang L, et al. Smoking and smoking cessation in relation to risk of diabetes in Chinese men and women: a 9-year prospective study of 0·5 million people. *Lancet Child Adolesc Health* 2018; published online March 13. http://dx.doi.org/10.1016/S2468-2667(18)30026-4.

Supplementary appendix

eTable 1. Adjusted HRs of incident diabetes by smoking status in men with additional exclusions or adjustments

eTable 2. Adjusted HRs of incident diabetes by amount smoked and adiposity in men

eTable 3. Baseline characteristics of women by smoking status

eTable 4. Baseline characteristics of male ex-smokers by reasons for, and years after, smoking cessation at baseline

eTable 5. Age-specific prevalence of diabetes in CKB versus age-specific prevalence in nationally representative surveys in China

eFigure 1. Adjusted mean adiposity levels by smoking status and daily smoking amount in men

eFigure 2. Adjusted HRs of incident diabetes by current regular smoking in men

eFigure 3. Adjusted HRs of incident diabetes associated with ever regular smoking by study area in men

eFigure 4. Adjusted HRs of incident diabetes associated with ever regular smoking by selected baseline characteristics in men

eFigure 5. Adjusted HRs of incident diabetes in male ex-smokers by reasons for, and years after (as a time-updating variable), smoking cessation

eFigure 6. Adjusted HRs of incident diabetes associated with ever regular smoking by selected baseline characteristics in women

eFigure 7. Adjusted mean adiposity levels in never smokers, current smokers and ex-smokers by years after cessation in men

eTable 1. Adjusted HRs of incident diabetes by smoking status in men with additional exclusions or adjustments

	Urban		Rural		Overall	
	No. of cases	HR (95% CI)*	No. of cases	HR (95% CI)*	No. of cases	HR (95% CI)*
**Excluding individuals with prior CVD and cancer at baseline**						
Never smokers	339	1.00 (0.89-1.12)	301	1.00 (0.89-1.12)	640	1.00 (0.92-1.08)
Ex-smokers	195	1.12 (0.97-1.28)	194	1.00 (0.87-1.15)	389	1.05 (0.95-1.16)
Occasional smokers	246	1.13 (0.99-1.28)	274	1.11 (0.98-1.25)	520	1.12 (1.03-1.22)
Ever regular smokers†	1293	1.22 (1.15-1.30)	1955	1.09 (1.04-1.14)	3248	1.15 (1.11-1.19)
p for heterogeneity		*0.018*		*0.35*		*0.014*
**Excluding diabetes cases during first 3-year follow-up**						
Never smokers	395	1.00 (0.90-1.11)	314	1.00 (0.89-1.12)	709	1.00 (0.93-1.08)
Ex-smokers	227	1.11 (0.97-1.26)	195	0.99 (0.86-1.14)	422	1.05 (0.95-1.15)
Occasional smokers	261	1.07 (0.95-1.21)	283	1.13 (1.00-1.27)	544	1.10 (1.01-1.20)
Ever regular smokers†	1412	1.17 (1.11-1.24)	2016	1.10 (1.05-1.15)	3428	1.13 (1.09-1.17)
p for heterogeneity		*0.052*		*0.20*		*0.023*
**Further adjusting for fruit and meat intake frequency**						
Never smokers	400	1.00 (0.90-1.11)	319	1.00 (0.89-1.12)	719	1.00 (0.93-1.08)
Ex-smokers	229	1.11 (0.97-1.26)	201	0.99 (0.86-1.14)	430	1.05 (0.95-1.15)
Occasional smokers	268	1.09 (0.97-1.23)	291	1.14 (1.01-1.28)	559	1.12 (1.03-1.21)
Ever regular†	1426	1.17 (1.10-1.24)	2060	1.10 (1.05-1.15)	3486	1.13 (1.09-1.17)
p for heterogeneity		*0.079*		*0.21*		*0.029*

* Stratified by age-at-risk and study area and adjusted for education, alcohol consumption, physical activity, body mass index and waist circumference.
† Ever regular includes participants who stopped smoking due to illness.

eTable 2. Adjusted HRs of incident diabetes by amount smoked and adiposity in men

	Never smokers		Cigarette or equivalent (g/day)					
			<20		20-29		30+	
	No. of cases	HR (95% CI)*	No. of cases	HR (95% CI)*	No. of cases	HR (95% CI)*	No. of cases	HR (95% CI)*
**BMI (kg/m^2)**								
<24.0	214	1.00 (0.87-1.15)	495	0.98 (0.89-1.07)	525	1.03 (0.94-1.12)	243	1.27 (1.12-1.44)
24.0-27.9	331	2.46 (2.20-2.74)	600	2.80 (2.59-3.04)	619	3.01 (2.78-3.25)	291	3.38 (3.01-3.79)
≥28.0	174	5.40 (4.64-6.29)	249	5.44 (4.80-6.17)	280	6.35 (5.64-7.15)	184	9.02 (7.80-10.43)
**Waist circumference (cm)**								
<85.0	268	1.00 (0.89-1.13)	559	0.90 (0.83-0.98)	584	0.92 (0.85-1.00)	264	1.11 (0.98-1.25)
85.0-94.9	276	2.31 (2.05-2.61)	524	2.64 (2.42-2.88)	521	2.66 (2.44-2.90)	248	3.01 (2.66-3.41)
≥95.0	175	4.67 (4.01-5.44)	261	4.64 (4.10-5.25)	319	5.64 (5.05-6.30)	206	7.49 (6.53-8.60)
**Body fat percentage**								
<25.0	366	1.00 (0.90-1.11)	690	0.91 (0.85-0.99)	689	0.94 (0.87-1.02)	315	1.14 (1.02-1.28)
25.0-29.9	319	2.31 (2.07-2.59)	596	2.53 (2.33-2.75)	640	2.74 (2.53-2.97)	345	3.53 (3.17-3.93)
≥30.0	34	3.56 (2.54-4.99)	58	3.48 (0.69-4.50)	95	5.65 (4.62-6.92)	58	7.05 (5.44-9.13)

* Stratified by age-at-risk and study area and adjusted for education, alcohol consumption and physical activity.

eTable 3. Baseline characteristics of women by smoking status

Characteristic	Never smokers (n = 270005)	Ex-smokers (n = 1049)	Occasional smokers (n = 5150)	Ever regular smokers (n = 7811)
Age (SD), years	50.1 (10.3)	61.0 (9.2)	51.9 (10.8)	58.7 (10.1)
Urban residence	118785 (44.2%)	530 (44.0%)	1946 (39.1%)	2772 (38.0%)
Education >6 years	119853 (44.1%)	244 (35.4%)	2276 (46.9%)	1862 (41.2%)
Exhaled carbon monoxide (SD), ppm	4.5 (4.9)	5.6 (7.8)	5.5 (8.6)	11.7 (26.4)
Random plasma glucose (SD), mmol/L*	5.7 (1.1)	5.7 (1.3)	5.7 (1.7)	5.7 (2.6)
Systolic blood pressure (SD), mmHg	129.1 (19.6)	126.2 (25.1)	127.1 (30.4)	125.4 (52.4)
Diastolic blood pressure (SD), mmHg	76.7 (10.6)	76.2 (15.1)	76.3 (16.4)	75.5 (31.1)
Weight (SD), kg	56.5 (8.7)	58.8 (12.8)	57.4 (14.6)	57.1 (24.7)
Height (SD), cm	154.2 (5.3)	154.7 (7.4)	154.5 (8.3)	154.9 (15.2)
BMI (SD), kg/m^2	23.7 (3.3)	24.5 (4.7)	24.0 (5.5)	23.7 (9.9)
Waist circumference (SD), cm	78.7 (8.9)	81.9 (12.2)	79.6 (14.5)	79.8 (30.1)
Waist-hip-ratio (SD)	0.9 (0.1)	0.9 (0.1)	0.9 (0.1)	0.9 (0.2)
Percent body fat (SD), % ‡	31.9 (6.8)	33.0 (10.2)	32.4 (11.2)	32.2 (21.0)
Physical activity (SD), MET-hr/day	20.8 (10.6)	17.7 (16.0)	19.7 (18.3)	18.4 (38.4)
Regular alcohol drinkers	5911 (2.4%)	186 (18.0%)	833 (11.7%)	1496 (12.2%)
Regular tea drinkers	57453 (21.0%)	169 (28.1%)	1141 (29.5%)	1805 (33.5%)
Regular meat consumption	118867 (43.9%)	408 (45.0%)	2249 (43.5%)	3123 (46.9%)
Regular fresh fruit consumption	86232 (32.1%)	414 (38.2%)	1778 (34.1%)	2079 (27.1%)
Family history of diabetes	18072 (6.6%)	44 (8.5%)	380 (6.8%)	406 (8.9%)

Values are means (SD) standardised to age and study area structure of the study population or number of participants (% standardised to age and study area structure of study population). BMI: body mass index; MET: metabolic equivalent task; Ex-smokers: ex-smokers who stopped by choice; Ever regular smokers: current smokers and ex-smokers who stopped due to illness.
* Data missing for 4681 participants.
‡ Data missing for 110 participants.

eTable 4. Baseline characteristics of male ex-smokers by reasons for, and years after, smoking cessation at baseline

Characteristic	Never smokers (n = 28214)	Ex-smokers stopped by choice			Ex-smokers stopped due to illness		
		≥15 years (n = 3388)	5-14 years (n =5807)	<5 years (n = 3755)	≥15 years (n = 2429)	5-14 years (n = 5417)	<5 years (n = 4612)
Age (SD), years	53.5 (11.9)	59.4 (10)	54.9 (10.7)	53.3 (11.1)	61.3 (9.2)	57.9 (10.4)	57.3 (10.4)
Urban residence	15278 (54.6%)	2094 (59.1%)	3021 (52.5%)	1600 (43.4%)	1350 (49.9%)	2439 (43.7%)	1648 (34.9%)
Education >6 years	18622 (61.4%)	2099 (62.0%)	3418 (59.8%)	2112 (58.4%)	1214 (57.7%)	2570 (56.6%)	1886 (52.8%)
Random plasma glucose (SD), mmol/L*	5.7 (1.2)	5.7 (1.3)	5.7 (1.3)	5.7 (1.3)	5.7 (1.6)	5.7 (1.3)	5.7 (1.4)
Systolic blood pressure (SD), mmHg	134.4 (20.2)	136.5 (22.7)	135.7 (20.5)	134.0 (20.4)	136.0 (24.2)	136.0 (21.9)	135.3 (23.6)
Diastolic blood pressure (SD), mmHg	79.6 (11.4)	81.0 (14.1)	80.4 (12)	79.6 (11.6)	80.8 (15.5)	80.2 (12.9)	80.3 (14.1)
Physical activity (SD), MET-hr/day	19.6 (13.0)	19.0 (16.1)	19.8 (12.7)	19.6 (12.5)	19.1 (19.4)	19.0 (13.9)	17.5 (13.0)
Regular alcohol drinkers	6627 (24.4%)	1618 (47.3%)	2905 (47.7%)	1690 (44.8%)	1200 (47.5%)	2688 (48.9%)	2250 (48.0%)
Regular tea drinkers	10166 (48.7%)	1536 (46.7%)	2890 (50.2%)	1758 (52.0%)	1115 (53.9%)	2536 (49.4%)	2102 (49.3%)
Regular meat consumption	15356 (51.7%)	1861 (53.6%)	3227 (55.6%)	1925 (55.8%)	1280 (54.3%)	2707 (54.6%)	2057 (53.0%)
Regular fresh fruit consumption	9310 (32.1%)	1236 (33.1%)	1811 (31.3%)	897 (27.3%)	818 (31.6%)	1553 (31.5%)	1004 (26.2%)
COPD	904 (8.3%)	140 (8.5%)	160 (7.8%)	110 (10.1%)	306 (16.2%)	572 (15.6%)	478 (16.3%)
Cardiovascular diseases	1588 (6.1%)	295 (6.1%)	278 (5.0%)	138 (4.4%)	271 (7.8%)	708 (12.1%)	709 (16.0%)
Hypertension	3619 (14.1%)	657 (15.5%)	810 (13.5%)	393 (11.1%)	552 (18.1%)	1241 (20.1%)	1065 (21.9%)
Cancer	127 (0.5%)	16 (0.3%)	25 (0.4%)	13 (0.4%)	42 (1.4%)	105 (1.7%)	175 (3.4%)
Family history of diabetes	1826 (6.1%)	222 (6.3%)	355 (5.9%)	212 (5.6%)	136 (7.2%)	343 (6.6%)	213 (5.4%)

Values are means (SD) standardised to age and study area structure of the study population or number of participants (% standardised to age and study area structure of study population). COPD: chronic obstructive pulmonary disease; MET: metabolic equivalent task.
Prevalence of prior diagnosed diabetes in CKB population at baseline: never smokers 4.0%; ex-smokers stopped by choice 4.5%; ex-smokers stopped due to illness 6.5%.
* Data missing for 2956 participants.

eTable 5. Age-specific prevalence of diabetes in CKB versus age-specific prevalence in nationally representative surveys in China

Age group (years)	Diabetes prevalence, % (95% CI)								
	CKB resurvey population (n=14,881)*			2010 China survey[†]			2013 China survey[‡]		
	Diagnosed[§]	Undiagnosed[‖]	Total[¶]	Diagnosed	Undiagnosed	Total	Diagnosed	Undiagnosed	Total
40-49	5.5 (4.8-6.1)	4.7 (4.1-5.3)	10.2 (9.3-11.1)	3.2 (2.9-3.4)	8.1 (7.7-8.6)	11.3 (10.8-11.8)	N/A	N/A	N/A
50-59	8.5 (7.7-9.3)	5.5 (4.9-6.2)	14.0 (13.1-15.0)	5.9 (5.5-6.2)	11.8 (11.2-12.3)	17.6 (17.0-18.3)	N/A	N/A	N/A
60-69	11.5 (10.2-12.7)	6.3 (5.4-7.3)	17.8 (16.3-19.3)	8.3 (7.8-8.9)	14.1 (13.4-14.9)	22.5 (21.6-23.4)	N/A	N/A	N/A
≥70	10.7 (8.2-13.2)	5.2 (3.2-7.1)	15.8 (12.8-18.9)	8.0 (7.3-8.8)	15.5 (14.4-16.6)	23.5 (22.3-24.7)	N/A	N/A	N/A
<40	2.7 (1.9-3.4)	2.5 (1.8-3.1)	5.1 (4.2-6.1)	N/A	N/A	N/A	1.3 (1.0-1.7)	4.5 (4.1-4.9)	5.9 (5.1-6.6)
40-59	7.1 (6.6-7.6)	5.1 (4.7-5.6)	12.2 (11.5-12.8)	N/A	N/A	N/A	5.0 (4.7-5.4)	7.8 (7.5-8.1)	12.9 (12.3-13.5)
≥60	11.3 (10.2-12.4)	6.1 (5.3-7.0)	17.5 (16.1-18.8)	N/A	N/A	N/A	8.8 (8.0-9.5)	11.4 (10.8-12.0)	20.2 (19.1-21.2)

[*] Participants included in both first and second resurvey.
[†] Xu Y, Wang L, He J, et al. Prevalence and control of diabetes in Chinese adults. JAMA. 2013;310(9):948-959.
[‡] Wang L, Gao P, Huang Z, et al. Prevalence and Ethnic Pattern of Diabetes and Prediabetes in China in 2013. JAMA 2017;317(24):2515-2523.
[§] Diabetes at baseline or incident diabetes (identified through disease surveillance system, health insurance system or death registries).
[‖] Self-reported or screen-detected diabetes in first or second resurvey, no diabetes at baseline and no incident diabetes (identified through disease surveillance system, health insurance system or death registries).
[¶] Diabetes at baseline, first or second resurvey or incident diabetes (identified through disease surveillance system, health insurance system or death registries).

eFigure 1. Adjusted mean adiposity levels by smoking status and daily smoking amount in men

Model 1 adjusted for age and study area. BF%: body fat percentage; BMI: body mass index; WC: waist circumference.

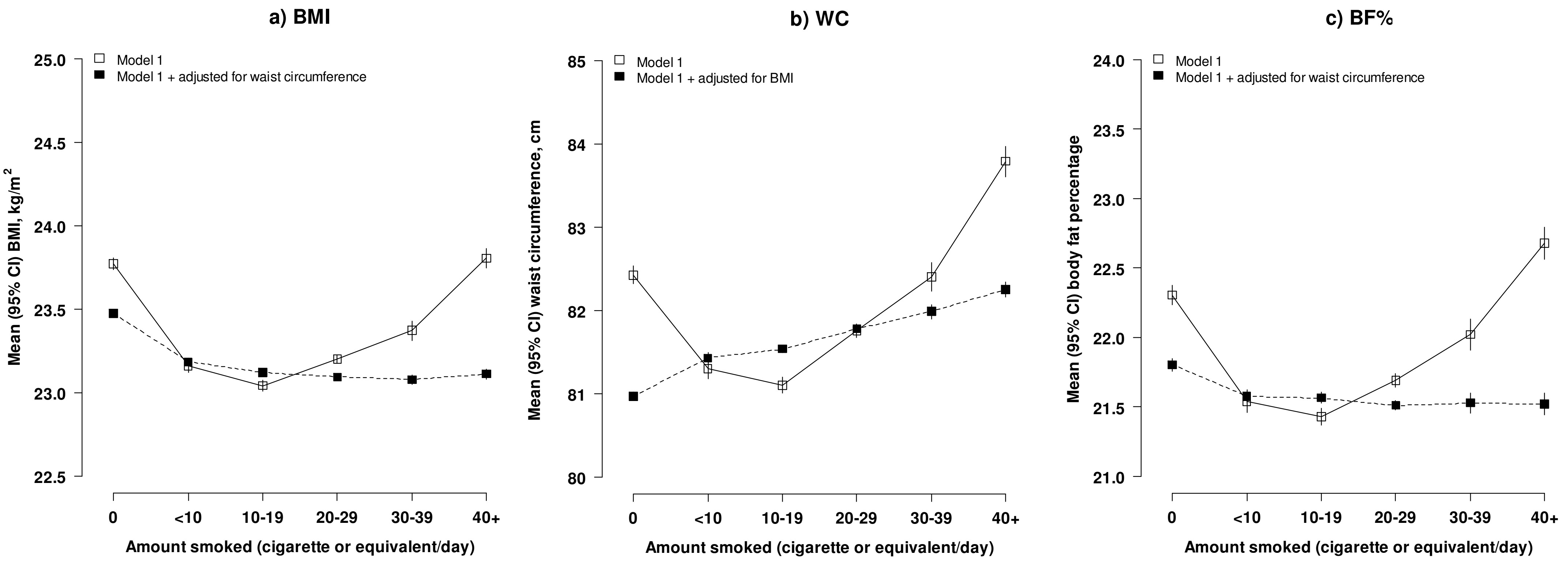

eFigure 2. Adjusted HRs of incident diabetes by current regular smoking in men

Stratified by age-at-risk and study area and adjusted for education, alcohol consumption, physical activity, BMI and waist circumference. Smoking duration analyses were additionally adjusted for age at baseline. Tests for trend included all smoking categories. Tests for heterogeneity included smokers only.

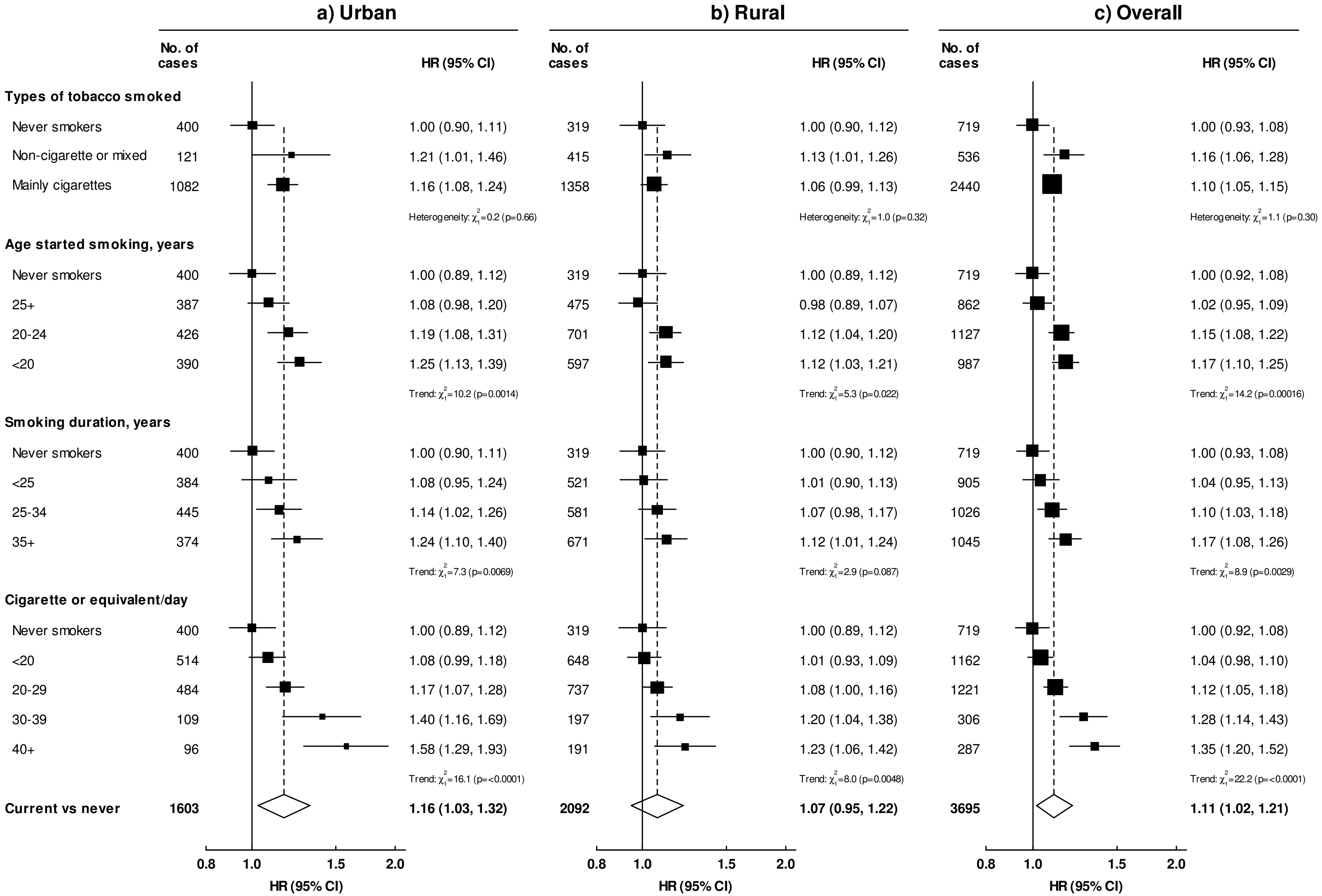

eFigure 3. Adjusted HRs of incident diabetes associated with ever regular smoking by study area in men

Stratified by age-at-risk and adjusted for education, alcohol consumption, physical activity, BMI and waist circumference.

	Ever regular smoking, %	No. of cases	HR (95% CI)
**Urban**			
Qingdao	65	214	1.20 (0.84, 1.71)
Harbin	58	474	1.19 (0.96, 1.48)
Haikou	47	179	1.15 (0.83, 1.57)
Suzhou	75	572	1.43 (1.05, 1.94)
Liuzhou	57	387	1.02 (0.80, 1.29)
			Heterogeneity: χ^2_4=2.9 (p=0.57)
**All**	**62**	**1826**	**1.19 (1.05, 1.34)**
**Rural**			
Sichuan	74	432	1.17 (0.88, 1.55)
Gansu	76	214	1.05 (0.71, 1.54)
Henan	66	183	0.82 (0.56, 1.22)
Zhejiang	74	1033	1.01 (0.84, 1.23)
Hunan	74	517	1.33 (1.03, 1.72)
			Heterogeneity: χ^2_4=5.1 (p=0.28)
**All**	**72**	**2379**	**1.10 (0.97, 1.24)**

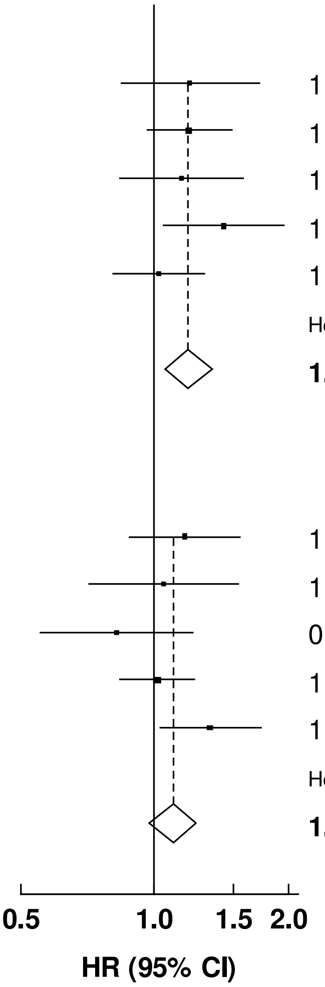

eFigure 4. Adjusted HRs of incident diabetes associated with ever regular smoking by selected baseline characteristics in men

Stratified by age-at-risk and study area and adjusted (except where it is the variable of interest) for education, alcohol consumption, physical activity, BMI and waist circumference. BMI: body mass index; MET: metabolic equivalent of task; SBP: systolic blood pressure.

	a) Urban		b) Rural	
	No. of cases (Ever/Never)	HR (95% CI)	No. of cases (Ever/Never)	HR (95% CI)
**Age-at-risk, years**				
35 to 54	447 / 70	1.35 (1.04, 1.76)	647 / 91	1.08 (0.86, 1.35)
55 to 64	534 / 120	1.12 (0.91, 1.39)	737 / 97	1.12 (0.91, 1.40)
65+	445 / 210	1.15 (0.97, 1.38)	676 / 131	1.08 (0.89, 1.31)
		Trend: χ^2_1=0.7 (p=0.42)		Trend: χ^2_1=0 (p=1.0)
**Educational attainment, years**				
<6	429 / 87	1.06 (0.83, 1.36)	1368 / 200	1.08 (0.93, 1.26)
6+	997 / 313	1.18 (1.03, 1.35)	692 / 119	1.13 (0.92, 1.37)
		Heterogeneity: χ^2_1=0.5 (p=0.47)		Heterogeneity: χ^2_1=0.1 (p=0.76)
**Physical activity, MET-hr/day**				
<10	453 / 184	1.15 (0.95, 1.38)	427 / 80	1.14 (0.89, 1.46)
10 to <20	405 / 119	1.23 (0.98, 1.52)	567 / 83	1.22 (0.97, 1.55)
20+	568 / 97	1.21 (0.96, 1.52)	1066 / 156	1.01 (0.85, 1.20)
		Trend: χ^2_1=0.2 (p=0.69)		Trend: χ^2_1=0.9 (p=0.33)
**Alcohol drinking**				
Never	191 / 135	1.06 (0.83, 1.34)	560 / 154	1.04 (0.86, 1.24)
Occasional	469 / 166	1.28 (1.06, 1.54)	521 / 82	1.22 (0.96, 1.54)
Regular	766 / 99	1.20 (0.97, 1.49)	979 / 83	1.11 (0.88, 1.39)
		Heterogeneity: χ^2_2=1.5 (p=0.47)		Heterogeneity: χ^2_2=1.1 (p=0.58)
**Fresh fruit intake frequency, day/wk**				
<4	482 / 196	1.24 (1.04, 1.48)	309 / 58	1.08 (0.81, 1.45)
4+	944 / 204	1.12 (0.95, 1.32)	1751 / 261	1.10 (0.96, 1.26)
		Heterogeneity: χ^2_1=0.7 (p=0.42)		Heterogeneity: χ^2_1=0 (p=0.92)
**Meat intake frequency, day/wk**				
<4	1029 / 297	1.16 (1.01, 1.34)	1026 / 131	1.23 (1.02, 1.48)
4+	397 / 103	1.22 (0.97, 1.55)	1034 / 188	1.01 (0.86, 1.19)
		Heterogeneity: χ^2_1=0.1 (p=0.72)		Heterogeneity: χ^2_1=2.5 (p=0.12)
**SBP, mmHg**				
<120	246 / 60	1.14 (0.84, 1.55)	329 / 40	1.15 (0.82, 1.61)
120-139	579 / 145	1.33 (1.09, 1.62)	853 / 124	1.17 (0.96, 1.42)
140+	601 / 195	1.12 (0.94, 1.33)	878 / 155	1.04 (0.87, 1.24)
		Trend: χ^2_1=0.3 (p=0.58)		Trend: χ^2_1=0.6 (p=0.44)

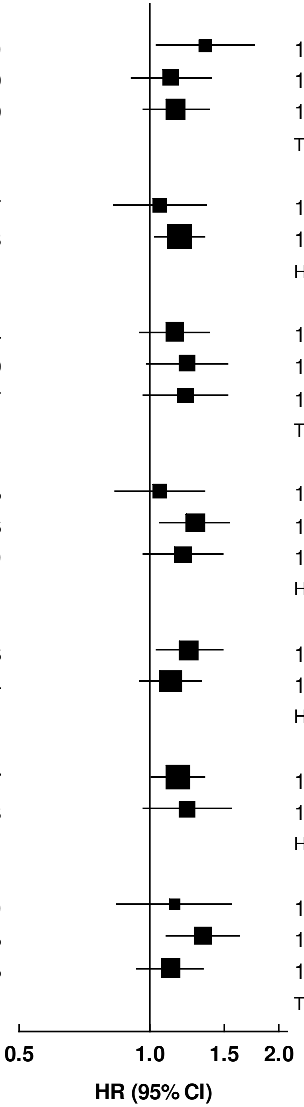

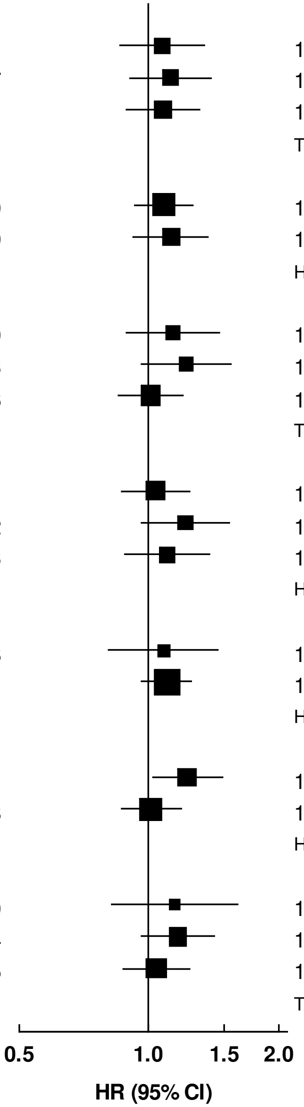

eFigure 5. Adjusted HRs of incident diabetes in male ex-smokers by reasons for, and years after (as a time-updating variable), smoking cessation

Model 1: stratified by age at risk and study area and adjusted for education, alcohol consumption and physical activity. Model 2: additionally adjusted for BMI and waist circumference. Tests for trend include only ex- smokers. BMI: body mass index.

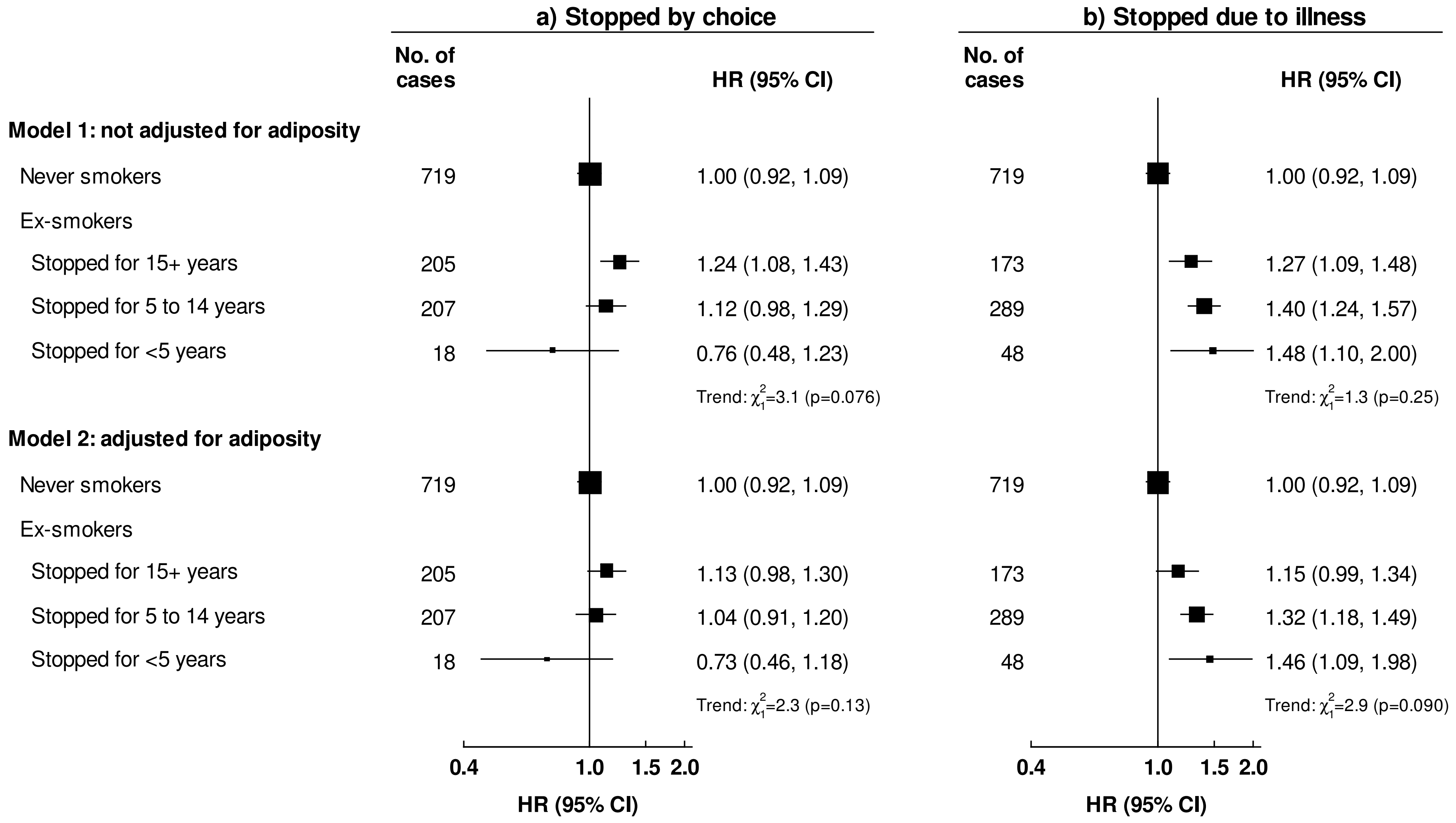

eFigure 6. Adjusted HRs of incident diabetes associated with ever regular smoking by selected baseline characteristics in women

Stratified by age-at-risk and study area and adjusted (except where it is the variable of interest) for education, alcohol consumption, physical activity, BMI and waist circumference. BMI: body mass index; MET: metabolic equivalent of task; SBP: systolic blood pressure.

	No. of cases (Ever/Never)	HR (95% CI)
**Region**		
Rural	262 / 4414	1.34 (1.16, 1.54)
Urban	111 / 3431	1.34 (1.10, 1.64)
		Heterogeneity: χ^2_1=0 (p=0.98)
**Age at risk, years**		
35 to 54	39 / 2264	1.54 (1.11, 2.13)
55 to 64	103 / 3115	1.29 (1.05, 1.60)
65+	231 / 2466	1.31 (1.13, 1.53)
		Trend: χ^2_1=0.4 (p=0.52)
**Educational attainment, years**		
<6	305 / 5467	1.35 (1.18, 1.54)
6+	68 / 2378	1.33 (1.04, 1.70)
		Heterogeneity: χ^2_1=0 (p=0.91)
**Physical activity, MET-hr/day**		
<10	118 / 1936	1.36 (1.11, 1.66)
10 to <20	133 / 2777	1.19 (0.98, 1.45)
20+	122 / 3132	1.49 (1.22, 1.83)
		Trend: χ^2_1=0.4 (p=0.52)
**Alcohol drinking**		
Never	200 / 5844	1.45 (1.25, 1.69)
Occasional	112 / 1842	1.14 (0.93, 1.40)
Regular	61 / 159	1.46 (1.03, 2.07)
		Heterogeneity: χ^2_2=3.7 (p=0.16)
**Meat intake frequency, day/wk**		
<4	150 / 3587	1.35 (1.13, 1.61)
4+	223 / 4258	1.33 (1.14, 1.54)
		Heterogeneity: χ^2_1=0.0 (p=0.90)
**Fresh fruit intake frequency, day/wk**		
<4	93 / 2349	1.26 (1.01, 1.57)
4+	280 / 5496	1.37 (1.20, 1.57)
		Heterogeneity: χ^2_1=0.4 (p=0.54)
**Baseline BMI, kg/m^2**		
<24	148 / 2576	1.34 (1.10, 1.62)
24 to <26	128 / 3334	1.30 (1.07, 1.57)
26+	97 / 1935	1.36 (1.09, 1.69)
		Trend: χ^2_1=0.0 (p=0.94)
**Waist circumference, cm**		
<80	187 / 4293	1.35 (1.14, 1.59)
80 to <90	114 / 2566	1.29 (1.06, 1.58)
90+	72 / 986	1.59 (1.22, 2.07)
		Trend: χ^2_1=0.6 (p=0.43)
**Body fat precentage, %**		
<30	45 / 512	1.45 (1.00, 2.10)
30 to <35	129 / 3158	1.32 (1.09, 1.61)
35+	199 / 4175	1.32 (1.13, 1.54)
		Trend: χ^2_1=0.1 (p=0.73)
**SBP, mmHg**		
<120	80 / 1509	1.57 (1.22, 2.01)
120-139	139 / 2830	1.30 (1.08, 1.57)
140+	154 / 3506	1.33 (1.11, 1.59)
		Trend: χ^2_1=0.8 (p=0.36)

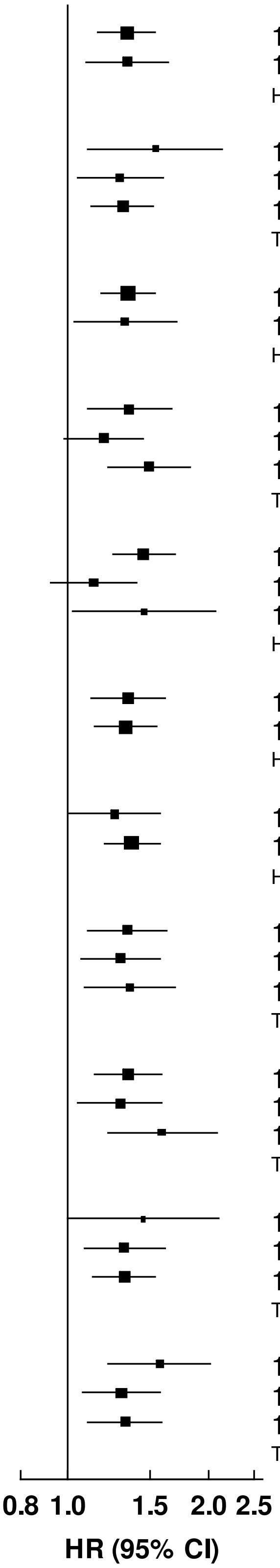

eFigure 7. Adjusted mean adiposity levels in never smokers, current smokers and ex-smokers by years after cessation in men

Adjusted for age and study area. BF%:body fat percentage; BMI: body mass index; WC: waist circumference.

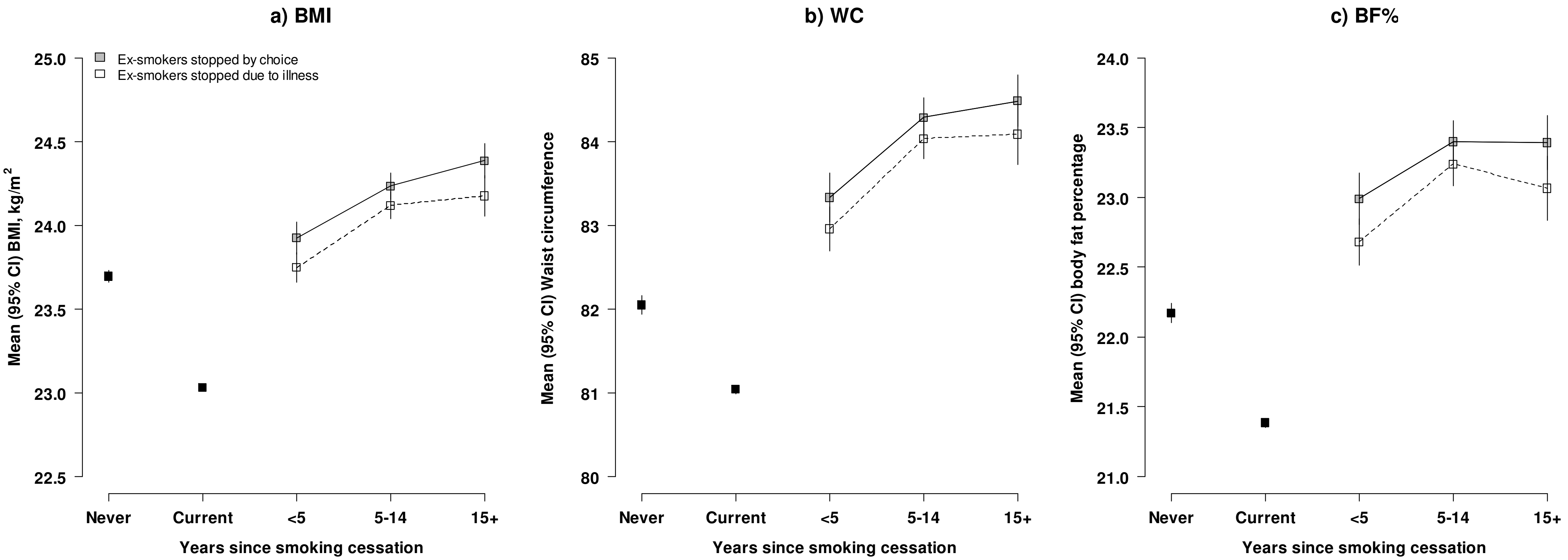

Supplement: Supplementary appendix [file mmc1.pdf]
